# Painful Times: The Emergence and Campaigning of Parents Against Injustice in 1980s Britain

**DOI:** 10.1093/tcbh/hwv024

**Published:** 2015-07-27

**Authors:** Jennifer Crane

**Affiliations:** University of Warwick

## Abstract

In July 1985 Steve and Susan Amphlett established Parents Against Injustice (PAIN) to support and represent parents falsely accused of child abuse. The Amphletts ran the organization from their own home, and struggled to gain funding, before closing PAIN in 1999. PAIN was to an extent a reflection of the ‘new politics’ of identity and lifestyle, concurrent with the rise of New Social Movements, as falsely accused parents utilized communication technologies to make their experiences public, and to contact and support one another. At the same time, PAIN also sought to exert political influence through relatively traditional channels—contributing to public inquiries, encouraging their membership to write letters to Members of Parliament, and shaping media critique. Despite its small size, PAIN was able to act as an intermediary between parents and politicians, social workers, solicitors and physicians. PAIN represented, but also collated and shaped, parents’ experiences. The case study of PAIN suggests that small groups have been able to mediate between ‘public’ and ‘experts’, effectively working with both groups because of their ability to combine experience and professionalism. These groups have brought experiential knowledge into social policy, and more broadly shifted the roles and responsibilities accorded to children, families and parents.

Parents Against Injustice (PAIN) was an advocacy group which, between July 1985 and November 1999, sought to represent parents falsely accused of child abuse. PAIN was founded by Susan and Steve Amphlett after the couple were mistakenly accused of physically injuring their youngest daughter.^1^ PAIN held the following ‘principal objectives’:

1. To campaign for reform in abuse and care procedures with accepted and enforceable codes of practice.
2. To ensure that the ‘principles of natural justice’ were given ‘due regard’ within these care procedures.
3. To encourage public debate on the rights of parents, children and families involved in child abuse cases.
4. To offer support and advice and to act as a referral helpline to parents unjustly accused of abuse.^2^

With these objectives, the organization served both as a lobbying group, seeking to influence policy-makers, care professionals, and media, and also as a support group, giving falsely accused parents emotional support and advice.^3^ In both its campaigning and its support work, PAIN mediated between falsely accused parents and physicians, lawyers, social workers and policy-makers. The group’s significance was recognized by contemporary social policy researchers, and relatively regular media coverage, and PAIN dealt with almost 13,000 cases over its lifespan.^4^ PAIN found influential patrons—Ludovic Kennedy, a writer and broadcaster with a keen interest in justice and false convictions and, later, Dame Margaret Booth, a High Court judge who had chaired the advisory committee on the *Children Act* of 1989.^5^

Despite these successes, PAIN was a very small organization. Archival material only exists for PAIN’s accounts in the financial years ending in April 1987, 1988 and 1989, although subsequent newspaper coverage and Parliamentary debates provide some indication of later accounts. From these sources, it is clear that PAIN relied on a sporadic and limited range of funding from public donations, government, charitable trusts and membership fees. PAIN’s income was just £4,428 in the financial year ending April 1987, £19,169 in 1988, and £52,528 in 1989—rising as the organization became more successful at seeking out grants from the Department of Health and various Trusts.^6^ PAIN was staffed full-time by Susan Amphlett, who was assisted by Steve Amphlett at the weekends and in the evenings, and also by between one and three secretaries as funding allowed. Secretaries engaged in a diverse range of tasks: filing, speaking to parents, organizing postage and photocopying, and drafting press releases.^7^

In 1996 the Department of Health substantially reduced the grants provided to PAIN, forcing the organization to fire its full-time employees. It is unclear why the Department stopped funding PAIN. Amphlett believed that the organization had lost momentum, and that prospective funders no longer regarded PAIN as ‘new and innovative’.^8^ Certainly, as I will describe later, new legislation brought parents further into child protection proceedings. Another journalist, Richard Ingrams, cynically suggested that: ‘It is, perhaps, not surprising that the State should cease to support an organisation which is trying to curb the excesses of state interference’.^9^ PAIN attempted to ‘stagger on’ with the support of a few volunteers and trustees, but the organization was struggling.^10^ In 1999 the Department of Health stopped funding PAIN entirely. Susan Amphlett told the sympathetic journalist David Brindle in 1999 that ‘We have tried every which way and there is simply nowhere left to make applications to’.^11^

In February 2000, Alison Stevens—a former volunteer for PAIN—restarted a new form of the organization, which has continued to offer support to parents and to release press and media statements.^12^ PAIN has not received formal funding since 1999, but has operated through a website, blogs, email and telephone.^13^ The group was still relatively influential—Stevens, for example, was labelled the ‘inspirational woman of the year’ by the *Mail on Sunday* in 2009—yet their reach was restricted by lack of resources. The first and second versions of PAIN were very different organizations, both of which merit academic attention. For example, examination of the second incarnation of PAIN could inform our knowledge of the relationship between the Internet and campaign organizations. However, this article will solely focus on emergence, campaigning and downfall of the original PAIN.

Whilst PAIN was a national group, local sites of parental activism also emerged in Britain in the 1980s and 1990s in response to widespread panics around child abuse in Leeds and in Cleveland and accusations of satanic ritual abuse in Orkney and Rochdale.^14^ National parents’ groups were also formed to campaign around specific issues, for example, The Five Percenters sought to defend parents falsely accused of shaking their babies.^15^ The local groups were very small—each representing between twenty and fifty local parents—and tended to only last for a couple of years when numerous accusations of child abuse were simultaneously made in particular areas. The Cleveland group, for example, was formed when two local paediatricians diagnosed 121 children, from fifty-eight families, as having been sexually abused, between late 1986 and Spring 1987.^16^ The judgement of the paediatricians was very controversial, as they relied on the new technique of reflex anal dilation, and indeed by 1991 ninety-four of the children had been returned to their families.^17^ During this case, forty-five of the accused parents were attending weekly meetings organized by a local clergyman, Reverend Michael Wright.^18^

Parent advocacy groups emerged, for the first time, as issues of child abuse and child protection began to accrue increasing levels of social and political concern from the 1960s.^19^ Before this time, there was some anxiety around ‘cruelty to children’, but this was seen as the responsibility of voluntary societies and philanthropists, rather than as an issue for all of society to prevent.^20^ In this context, social services and physicians were increasingly looking to identify cases of child maltreatment but, as Ian Hacking has written, to be accused of child abuse was to be accused of committing the ‘worst possible vice’.^21^ It is impossible to find statistics on the number of false accusations, but parents groups believed that there were many within this ‘anxious climate’.^22^

PAIN was thus the largest group within a broader movement towards parental activism. Phillip Jenkins and Nigel Parton have both paid brief attention to PAIN, and written that the group achieved ‘striking success in presenting its views to the mass media’, and was the ‘most coherent voice’ in ‘the parents’ lobby’.^23^ Aside from this, however, the organization has not yet been subjected to sustained academic attention. In part, this is perhaps because PAIN was a short-lived and also relatively small organization. Writing a history of PAIN is also complicated by a lack of available resources. When PAIN closed, the Amphletts destroyed the group’s case files, containing the demographic details and histories of the PAIN families, reflecting late-twentieth-century concerns around familial privacy and secrecy, as well as a cathartic, or perhaps frustrated, closure to the campaigning careers of the Amphletts.^24^ No archives hold a substantial body of PAIN’s published materials. I have traced the history of PAIN through a disparate range of sources: newspaper articles, a television documentary, submissions to public inquiries, published materials, and four of the groups’ newsletters available at the British Library. From these materials, this article argues that PAIN provides a useful lens through which to study the nature of expertise, voluntary action and policy amidst the changing construction of respectability, parenting and state in late-twentieth-century Britain.

Frank Prochaska has claimed that civic participation in Britain declined in the post-war period, in comparison to the ‘golden age’ of the Victorian era.^25^ Matthew Hilton, James McKay, Nicholas Crowson, and Jean-Francois Mouhot have persuasively countered this thesis by arguing that a change has occurred in the nature, not the extent, of civic participation, as the post-war period saw the passive membership of non-government organizations replace the active membership of political parties, trade unions, and churches. The membership of non-governmental organizations can be mobilized at key junctures of crisis, but the public are more likely to support a group through arms-length, ‘cheque-book activism’ than to attend regular meetings or to actively campaign.^26^ The case study of PAIN demonstrates that, to some extent, a tradition of small, locally run, informally organized activism continued to be influential in the post-war period.

Furthermore, I will argue that PAIN was shaped by a mesh of old and new politics and forms of voluntarism. PAIN drew upon the ‘new politics’ of identity and lifestyle manifested in the New Social Movements of the 1960s and 1970s but, at the same time, also encouraged parents to help one another, reminiscent of older ‘self-help’ movements. Also, PAIN sought to influence policy-makers in a relatively ‘traditional’ manner, seeking negotiation and consensus from within the system. My argument draws upon Alex Mold’s characterization of Release, an organization established in 1967 to provide legal assistance to people arrested for drug offences. Mold argued that Release ‘by concerning itself with questions of identity and lifestyle was representative of an interest in new types of political and social problems, but these were often resolved by using old political strategies’.^27^ Mold suggests that Release was a ‘buffer’ between the alternative society of the 1960s and ‘the establishment’.^28^ In this article, I wish to further examine the notion of the ‘buffer’, and to understand why and how voluntary groups were able to perform this role. I will argue that groups are able to work with factions of the public, mobilized by identity, and also with physicians, social workers, media and policy-makers because of their ability to combine personal experience with professional expertise. First, I will demonstrate that PAIN was able to encourage falsely accused parents to speak about their traumatic experiences, for the first time, because the groups’ publicity materials described the life histories of the Amphletts. At the same time, PAIN was able to establish structures through which parents could help one another, such as local support groups, letter exchanges and a helpline, which united parents and also demonstrated relatively high levels of organization. Second, this article argues that PAIN was able to influence policy-makers because the organization could claim to hold experiential knowledge, as policy-makers increasingly sought out the experiences of patients and service-users in the late twentieth century.^29^ At the same time, PAIN influenced policy-makers through traditional channels—writing to Members of Parliament, contributing to public inquiries, and speaking to the media. In doing so, PAIN played a role in bringing the parental perspective into social policy, changing the balance of rights and responsibilities between parents and children. Third, I will question the extent to which PAIN could effectively represent the ‘experiences’ of all falsely accused parents. The limited evidence available suggests that PAIN spoke primarily for middle-class parents, and PAIN certainly emphasized a representation of their membership as ‘respectable’ two-parent, mother-oriented families. Whilst Hilton et al have highlighted the significance of professionalization in guiding the post-war voluntary sector, I will assert that experiential knowledge has also been significant, and that these two, apparently distinct, forms of knowledge have in fact been combined and entwined within small voluntary organizations.

## PAIN and Parents

PAIN worked effectively with falsely accused parents because the Amphletts themselves had had personal experience of the child protection system, as had many of PAIN’s other staff and volunteers. In their support work, PAIN sought to enable parents to communicate with one another, a project which assumed the value of shared experience. To facilitate these support networks, PAIN instated formal structures of management and organization, reflecting the broader ‘professionalization’ of the voluntary sector. Thus, parents came to PAIN because of the Amphletts’ personal experiences but, at the same time, relied upon PAIN’s expertise in establishing channels for contact, gaining funding, and communicating with solicitors, physicians and social workers.

When describing their reasons for establishing PAIN, the Amphletts focused heavily on their personal history. One undated pamphlet, simply labelled ‘Publicity Material’, told the Amphletts’ story as follows: Between October 1983 and February 1984 the Amphletts’ youngest daughter sustained two fractures on her right arm.^30^ After her second fracture, the Amphletts took her to an accident and emergency ward. There, they were referred to social services. A family case conference was held to establish whether the children were safe living in their home. The Amphletts’ daughters were not taken into care, but the children were placed on the Child Abuse Register, a list of children considered at risk of abuse, and Susan was cautioned by police officers.^31^ Nine months after this case conference, following sustained monitoring by social services, the Amphlett children were removed from the Child Abuse Register, but shortly afterwards the youngest daughter sustained another fracture.^32^ On seeking further medical advice, Susan and Steve found that she had brittle bone disease, explaining why she had always sustained fractures after relatively minor falls.^33^ Susan and Steve stated of their experiences that: ‘[we were] appalled by the manner in which we have been treated’, and particularly by the fact that they were not allowed to attend the case conference convened to discuss their daughter.^34^ Hoping to help other parents who had been falsely accused of child abuse, the Amphletts founded PAIN. Steve and Susan’s account was thus confessional, as the Amphletts explained that, like other parents, they too had been accused of fundamentally transgressing social norms, and abusing their children. Their account was also inspirational, as the Amphletts explained how they had proven their innocence, and not lost custody of their children. By placing their experiences at the forefront of their campaigning literature, the Amphletts could reassure other falsely accused parents that they understood their experiences and could provide support.

Steve was an engineer by trade, and Susan a nurse, which were not professions directly equipping them to organize a voluntary group. Nonetheless, Susan became PAIN’s Director, whilst Steve continued to work as an engineer and to help with PAIN in the evenings and at weekends. PAIN’s work was primarily carried out from a small office in the Amphletts’ home in Bishop’s Stortford, Hertfordshire.^35^ Alongside Susan, the office was staffed by between one and three secretaries, at various points, and in PAIN’s newsletter of August 1988, a temporary secretary Liza described their home as: ‘A semi-detached bungalow in the middle of nowhere, under the flight path to Stanstead [sic] airport … The dining table was covered with heaps of paper with a large typewriter half buried in the middle. . . Boxes of papers created an obstacle course across the floor’.^36^ She wrote that temps ‘get jobs in odd places … but this was the most unusual’.^37^ Thus, the article continued, to work in such a site: ‘These people were obviously dedicated to the work of the charity’.^38^ A contribution to the August 1988 newsletter by PAIN’s secretary Noelene similarly emphasized the dedication of the Amphletts, stating that ‘I have not just joined a Charity but a Family … a very loving and caring family’.^39^ The Amphletts allowed the support work of PAIN to pervade their home and free-time, and to redirect Sue’s career.

Throughout its lifespan, PAIN conducted support work with numerous parents. PAIN took parents’ protestations of innocence at face value, because of their recognition that innocence was sometimes difficult to prove, and their belief that many parents were being falsely accused.^40^ PAIN’s willingness to represent any parent was occasionally, though not regularly, challenged. The *Guardian* journalists Victor Smart and James Erlichman suggested that PAIN could be utilized to ‘provide respectable cover for child molesters’ in 1987 and 1997—demonstrating that complaints about the groups’ legitimacy were continuing throughout its lifespan.^41^ Nonetheless, between July 1986 and December 1987 PAIN had advised over 1,000 families, and had conducted sustained case work with 500 of these. In 1998, Susan Amphlett wrote that the organization advised and supported approximately 900 new families annually, and remained involved in a further 2,500 ongoing cases.^42^ Susan Amphlett wrote in PAIN’s July 1987 newsletter that ‘the time spent on each history obviously varied’ between 11 and 70 hours, and would involve phone calls to support the parents, phone calls to explain, mediate and facilitate the relationship between the parents and their solicitor, doctors and psychiatrists, home visits on some occasions, and the production and maintenance of PAIN’s records.^43^

To carry out this kind of case work, the Amphletts developed in-depth technical knowledge of the child protection system. PAIN offered professional expertise in a supportive and understanding manner, identifying and working with individual professionals who were sympathetic to their cause. For example, PAIN put many families in touch with Dr. Colin Patterson, a physician who invented the controversial category of Temporary Brittle Bone Disease, and between 1975 and 1987 intervened in fifty child protection cases to diagnose the disease.^44^ The workers at PAIN had acquired high levels of professional knowledge but, due to their limited staff-base, PAIN struggled to maintain their case load. In the newsletter of July 1987 Amphlett apologized ‘for the delay some of you may be experiencing in getting assistance from us’.^45^

PAIN was also able to establish regional branches, to ease the workload of the Headquarters.^46^ The first branches were established in Leeds, Oldham, Blackburn and Wales.^47^ In subsequent years, branches were founded in London, the East of England, the South and South West, the North of England, Scotland, the Midland region and the North East.^48^ The services which each branch provided varied, dependent on the availability of the ‘Regional Administrators’, but all offered telephone and letter-writing support, and some established support groups for local parents.^49^ Referencing a new support group in the August 1989 newsletter, the Administrator for the East of England wrote that ‘These families have a lot in common and have been in contact and found each other very supportive’.^50^ PAIN gave some funding to their regional centres—£6,570 in the financial year ending 1989—but Administrators were also expected to raise their own income to cover their telephone and postage bills.^51^

The majority of Regional Administrators came to the role having themselves been helped by PAIN. Alison Stevens, for example, was a long-serving Regional Administrator for Leicestershire, working for PAIN from 1986 until 1999. Stevens was falsely accused of abusing her 3-year-old son in 1985, and a *Leicester Mercury* profile of 2005 stated that after recovering her child ‘Alison decided to use her experience to help other parents in a similar situation’ and also quoted Stevens’ assertion that ‘I was quite lucky to have someone to help me and now it helps me help others—they get to talk to someone who understands and has been through it’.^52^ However, not all Administrators had themselves been falsely accused of abuse: the representative for the Midlands Region wrote in PAIN’s 1989 newsletter that she was ’an ordinary mother’ but as a children’s nurse had ‘grown more concerned about the way children can so easily (it appears to me) be removed from their home on POSO [Place of Safety Order]’.^53^ Administrators were thus always passionate about PAIN’s cause, but had been brought to PAIN by different experiences.

Seeking to disseminate her knowledge, Susan Amphlett trained the Administrators herself, providing advice on counselling, fundraising and publicity.^54^ Administrators described the training as ‘intensive, rigorous yet enjoyable’ and leaving them ‘“shell-shocked” in more ways than one!’^55^ Indeed, the Administrator role was difficult and time-consuming, and Administrators often wrote to PAIN’s newsletter to apologize for being temporarily unable to receive telephone calls or to ‘remember your name or the details of your particular case’.^56^ Administrators also described their work as emotionally draining, with one writing that ‘I wonder if the phrase “fools step in where angels fear to tread” is a good description of me these days!’ and another that ‘I feel your hurt, anger and despair’.^57^ Administrators sought to find local representatives within their regions, but were not always successful, and there was a high turnover within this role.^58^

PAIN thus was built on experience on both a national and a regional level but, at the same time, needed to act professionally in order to handle its heavy case-load. The entwinement of professional and experiential knowledge may be seen in the composition of PAIN’s Board of Trustees, which was composed of between four and nine Trustees, and met forty times between 1985 and 1998.^59^ Throughout PAIN’s existence, Trustees were a mix of Regional Administrators, parental activists, and representatives from medicine, social work, and the law.^60^ PAIN’s funding, also, was drawn from both professional bodies and also from supportive parents. In 1987, 92 per cent of PAIN’s £4,428 funding was drawn from its supporters and the public, and the remaining 8 per cent generated from loans, interest on the deposit account and cash already in hand.^61^ After PAIN registered as a charity in 1987 the organization also began to receive grants from the government and charitable Trusts. In the financial year ending 1989, for example, PAIN gained grants from the Department of Health (£18,000), the Mental Health Foundation (£10,000), Telethon Trusts (£5,500), The Fairway Trust (£5,000), The Hilden Trust (£1,000), the Charles French Trust (£200), the Edward Cadbury Trust (£1,000), the Noel Buxton Trust (£1,000), and the Courtald Trust (£500). Until 1996 the government continued to provide PAIN with annual grants of between £10,000 and £35,000.^62^

PAIN also continued to gain funding from a loyal support-base of involved parents. PAIN gained money from their membership fee—a £10 annual charge—and from fund-raising activities organized by members, including marathon running in Bolton and a Fair Day in Newcastle on Tyne.^63^ Parental fundraising raised relatively substantial sums: £7,169 in the financial year ending 1988, and £8,528 in 1989. Still, newsletters emphasized that the group was in ‘desperate need’ of more funding, and stated that: ‘we really have achieved an incredible amount on a very small sum of money. Imagine how much more effective we could be in [sic] we had reasonable funding’.^64^

Susan Amphlett described PAIN as a ‘self-help organisation’ in the group’s first newsletter.^65^ Providing a preface to this newsletter, the social work academic Nigel Parton wrote that PAIN ‘is not a professional group with its own professional interests to advance. It is a group of parents who have come together primarily because of similar experiences. The role of providing mutual support and advice is vital. There are very few places people can go to for this’.^66^ PAIN was not only a self-help group, but created formal structures such as a Board of Trustees and patrons, as well as compiling audited accounts, training their Administrators, and employing some full-time staff. Nonetheless, PAIN’s publicity materials regularly reiterated that the organization had been formed because of personal experience, and PAIN did seek to create structures through which falsely accused parents could help one another, particularly through the phone line, support groups, and the system of Regional Administrators. PAIN was able to work effectively with parents because it utilized and emphasized experiential knowledge. In working with parents, however, the leaders of PAIN mediated between parents and solicitors and physicians and social workers, and themselves became ‘experts’ in this area, their own knowledge-base stretching beyond personal experience alone.

## PAIN in Policy

PAIN also mediated between falsely accused parents and policy-makers, seeking to bring the experiences of parents into the construction of policy. PAIN offered case studies to newspapers and public inquiries, and encouraged parents to write directly to their Members of Parliament. PAIN was, to an extent, working within the political system and bringing experience into policy through the mainstream channels but, at the same time, was also very critical of the state in many of its statements to newspapers and parents.

PAIN provided case studies to left-wing, right-wing, tabloid and broadsheet newspapers, which were reproduced by journalists including David Brindle from the *Guardian* and Anthea Gerrie from the *Daily Mail*.^67^ PAIN also gave a series of case studies to the Inquiries around the cases in Cleveland, Rochdale and Orkney, and also submitted a response to *Child Abuse—Working Together, A Draft Guide to the Arrangement for Inter-Agency Co-operation For the Protection of Children*, a booklet produced by the Department of Health and Social Security in 1986.^68^ The case studies provided were usually concise, and explained the situation of the family, their background, the child protection intervention, and the course of action which was eventually taken. A case study published in the *Daily Mail* in June 1987 was as follows:

ENGINEER Peter (not his real name) and his wife Paula had taken their five-year-old mentally retarded son to hospital for a general consultation, when the shocking diagnosis came back of sexual abuse by the child’s father.

The little girl was not allowed home but spent her sixth birthday and Christmas in the hospital before being transferred to a children’s home. It took three nightmarish months before independent assessments, asked for not only by the parents but also by the court, proved that the diagnosis was totally without foundation. This hospital consultant had made the diagnosis following a secret session with the child in which he asked her to play with ‘anatomically correct’ dolls.^69^

This case study represents several themes common to those offered by PAIN. First, the idea that parents had openly taken their child to a physician.^70^ PAIN’s fifty-page response to *Child Abuse—Working Together*, similarly, stated that ‘Our parent members are the type of people who use the services available such as clinic, health centres and casualties ... Their babies and children are taken regularly to be weighed and examined and are up to date with their vaccinations’. PAIN’s members were the ‘type of people’ who diligently utilized state-provided education, health and welfare, a representation which chimed with the contemporary work of Michel Foucault around how the experts and agents of ‘the state’ observed, governed and disciplined families along lines of ‘normal’ and ‘abnormal’ conduct.^71^

Second, case studies often emphasized the devastation of parents caught up in this ‘hellish’ or ‘nightmarish’ situation.^72^ Parents utilized highly emotional language, describing their ‘anguish, anxiety, shame, helplessness’, ‘fear’, ‘anger’, ‘disbelief’, ‘despair’, ‘horror’, ‘terror’, ‘helplessness’ and ‘sheer desperation’.^73^ The organization’s acronym, PAIN, also emphasized the emotions felt by falsely accused parents.^74^ The emotional language underlined the distress for dedicated parents who were unable to perform the ‘normal’ activities of familial life, such as tucking their children into bed, reading bedtime stories and being present for birthdays.^75^ Third, case studies tended to revolve around relatively clear-cut cases.^76^ The *Mail’*s case was shown to be ‘totally without foundation’, and other cases found children who had been proven to have brittle bone disease, or where medical practitioners had mixed up slides or diagnoses.^77^ Thus, PAIN was emphasizing the cases where parents’ innocence was conclusively proven.

PAIN sought to emphasize that each of these case studies were not isolated incidents, but rather the ‘tip of the iceberg’ under which a mass of families were being wrongfully accused of child abuse. The organization often offered statistics to support this assertion, for example in telling the *Guardian* that six out of seven of the 160,000 children annually subjected to child abuse investigations had not been abused, and were not ultimately placed on the child abuse register.^78^ With the evidence of case studies and statistics, PAIN then made policy suggestions, calling for the creation of a complaints procedure for parents, the improved collection of statistics around the incidence of child abuse, and a guarantee that parents would have the right to be assessed with their children in their own homes, and to gain a secondary medical opinion.^79^

PAIN thus mediated parental experiences by choosing case studies, and drawing particular lessons from them. PAIN was able to bring these collated experiences to the media and public inquiries because of their professional expertise, which enabled them to build relationships with specific journalists and policy-makers, and to write persuasively. Indeed, in her statement to the Cleveland Inquiry, Susan Amphlett opened by asserting that: ‘I am a fully qualified State Registered Nurse and hold a Part I Central Mid-Wives Board, Post Registration Course in Occupational Health Nursing Certificate with Distinction’.^80^ When talking to policy-makers, Amphlett emphasized her traditional forms of expertise. With this in mind, she continued: ‘As a result of my own experience … I have become concerned’, before outlining several case studies and conclusions.^81^

As well as collating and representing parents’ experiences, PAIN also encouraged its parents to write directly to Members of Parliament. Amphlett wrote in PAIN’s first newsletter that ‘We must inform our MPs of what is happening and we must complain to our local councillors. We must complain in writing, to Directors of Social Services and we encourage you to use your stories to illustrate bad practices whenever possible’.^82^ Janet Ali, one of PAIN’s Trustees, also advised in the August 1988 newsletter to ‘write to local MPs and particularly local Councillors’. From her experiences as a solicitor, Ali noted that these actors ‘can be very helpful. Directors of Social Services usually take note of complaints by the local Councillors and MPs’.^83^ The circulation of PAIN’s newsletter was 1,200 in 1988, although there is no way to trace how many parents took up this call.^84^ Nonetheless, it is significant that PAIN believed in the ability of ‘stories’ to ‘illustrate’ bad practice, and to inform change, and this pays testament to the confidence of the voluntary sector in the significance of experiential knowledge in the late twentieth century.^85^

Whilst PAIN sought to work with policy-makers to inform change, the organization was also, sometimes, highly critical of the state. In a pamphlet from 1986, provided to parents, PAIN suggested that ‘the authorities’ acted to trick innocent parents into confessing their guilt with threats and coercion. A section entitled ‘Things the Authorities Say’ included ‘If you try to get your husband out on bail you will never see your baby again’, ‘If you admit to hurting your baby you will get him back sooner’ and ‘Even if you haven’t done it, it would be better if you say you have’.^86^ PAIN was also very critical of state power when speaking to the media. Amphlett claimed that the state should not be a parent, taking children into care to raise itself, and leaving ‘real’ parents’ ‘powerless and helpless’, a potent idea amidst the growing awareness of institutional abuse in the 1990s.^87^ Drawing on the Thatcherite notion that the economic extension of the state was related to moral decline, Amphlett asked: ‘What damage are we doing to the structure of our society? What damage are we doing to family life?’^88^ In 2000, Amphlett drew on the language of empowerment, telling the *Guardian* that: ‘The system is like a huge juggernaut which rides over whatever you try to do. It is very alien to most parents and leaves them feeling disempowered’.^89^

Whilst PAIN sought to criticize child protection, its arguments were taken to denounce the overextension of *all* state power, and in 1996 PAIN was described in the *Guardian* and the *Observer* newspapers as ‘an organisation which is trying to curb the excesses of state interference’ and ‘an organisation which fights state excesses and which actually *knows* the state does harm’.^90^ The *Daily Mail* also utilized the case study of PAIN to make a broader critique. In a 1987 article entitled ‘How can they call this “care”?’, Gerrie wrote:

Like the Pol Pot regime of Cambodia, we have become chillingly adept at separating children rapidly from their parents and placing them under State rule, through the official powers we have awarded ourselves. There is nothing to equal the harm done to children by sex abuse, but it is our concern with this that perhaps blinds us to the awful cruelty that the State can inflict upon innocent children.^91^

Dramatically, Gerrie utilized case studies offered by PAIN to suggest that the British state was as overextended as the tyrannical regime of Pol Pot. To an extent, by criticizing ‘the state’, PAIN was echoing the language and experiences of individual parents, and contemporary social surveys found that parents who had encountered child protection services often tended to perceive all state workers in child protection as the same, generic ‘professionals’ who talked down to parents with a ‘we know best’ attitude.^92^

Thus, PAIN sought to bring parents’ experiences into policy through traditional channels and also through more radical critique, and PAIN sometimes reflected the way in which some parents criticized ‘the state’ and professionals, and sometimes encouraged parents themselves to contact their Members of Parliament and to work within ‘the system’. The extent to which PAIN was successful in bringing the experiences of falsely accused parents in to policy is difficult to ascertain. Certainly, the *Children Act* of 1989 stated that it was ‘a charter for children’ but, at the same time, that: ‘Central to the philosophy of the Act is the belief that children are best looked after within the family with both parents playing a full part and without resort to legal proceedings’.^93^ In terms of child protection, the Act replaced Place of Safety Orders with Emergency Protection Orders (EPOs). Both were mechanisms through which social workers could remove children from their parents or guardians. However, parents could not challenge POSOs for 28 days, whereas EPOs could only last for 8 days (unless extended by the court for an additional week), and parents could challenge these orders after just 72 hours.^94^ The Act also mandated that authorities would ‘seek the views’ of parents when child protection cases were being reviewed, and would also ‘notify details of the result of the review and of any decision taken by them’.^95^ Local authorities now had to establish a procedure to allow parents to complain, and to ensure that at least one person who was not a member of the authority took part in case reviews.^96^

PAIN was not satisfied by the changes made to policy. On the organization’s closure in 1999, representatives of PAIN wrote to the *Guardian* to bemoan that ‘things had not improved’ since the groups’ foundation in 1986.^97^ In 2009 Trevor Jones, on behalf of the reconstituted PAIN, told a public inquiry that the organization had seen ‘very little change for the better’ and was still ‘advising and advocating on the same issues’.^98^ Jones reiterated this conclusion when providing written evidence to the House of Commons Education Committee in 2012.^99^ We must not overstate the extent to which parents have been incorporated into the child protection intervention since the 1980s. Nonetheless, it is significant that policy-makers have sought to include parents, and also to pay testament to the need to hear parents’ voices in both the policy-making process and the implementation of child protection work. Notably, the Conservative Party manifesto of 1997 echoed the arguments and rhetoric of PAIN in stating that: ‘A heavy-handed and intrusive state can do enormous damage … When the state goes too far, it is often the children who suffer’.^100^

The ideas espoused by PAIN thus became influential in later years. Some contemporary commentators suggested that PAIN had influenced this shift, by bringing the experiences of parents to the attention of policy-makers. A practitioners guide from 1991, *The Child Protection Handbook*, stated that PAIN was ‘influential in ensuring that the rights of parents and of children to be left at home, free of state intervention and removal, were placed on the political and professional agendas’.^101^ On PAIN’s closure, Chris Davies, the President of the Association of Directors of Social Services, reflected that the existence of PAIN had shown that social services must find ‘ways of parents having an input’.^102^ In 2000, the Chairman of this Association’s Children and Families Committee, Rob Hutchinson, also stated that PAIN was ‘very challenging to us’ and ‘helped maintain a balance as parents’ advocates in the hugely difficult area of child protection’.^103^

At the same time, many other factors were at play: contemporary policy researchers simultaneously argued that parents should have more of a say in child protection, the local authority of the London Borough of Sutton was experimenting with this approach, and prominent public inquiries reached similar conclusions.^104^ The theorist of social work Ray Jones has argued that the Children’s Acts produced between 1948 until 2008 were driven by both policy research and changes in practice.^105^ However, Jones does not also recognize the significance of campaign groups. Furthermore, changes in policy research, practice and the voluntary sector were entwined with one another as particular individuals worked between and within each of these fields. The critique which PAIN made through the media was viewed by social workers, policy-makers, and other charities, some of whom directly responded to PAIN in the letter pages of newspapers.^106^ PAIN drew lessons from and advised the Cleveland Parents Support Group, who in turn provided evidence to the Butler-Sloss inquiry. Mr David Monk, the Area Manager of Sutton Social Services, became a Trustee of PAIN in 1988, as did Reverend Michael Wright, who organized the group at Cleveland.^107^ Groups such as PAIN brought experiential knowledge into policy, and to the attention of social workers, physicians and public.

## The Limits of ‘Experience’

PAIN thus mediated between falsely accused parents, policy-makers, solicitors, physicians and social workers. PAIN was able to work with each of these groups because the organization sought to represent personal experience in a professional manner. But did PAIN represent the experiences of all falsely accused parents? I will demonstrate that the evidence available suggests that the majority of PAIN’s membership were middle class. Furthermore, I will argue that PAIN worked hard to actively represent their membership as ‘respectable’; articulate, affluent, and mother-oriented families. By propagating this representation, PAIN drew on broader anxieties about the state of the family, and argued that its members were not the ‘type of people’ who would abuse their children. Whilst this presentation reflected prevailing social concerns, it also placed clear limitations on the ability of the group to represent all falsely accused parents.

It is impossible to know the demographics of the families who PAIN worked with, as their case files were destroyed. It is likely that the families involved in PAIN were what may be characterized as ‘middle class’. In a study conducted during the late 1980s and early 1990s, the sociologist Jon Prosser visited the homes of thirty parents falsely accused of abuse and represented by PAIN. Prosser stated that ‘middle class parents’ embroiled in child protection interventions were more likely than their ‘working class counterparts’ to obtain a lawyer earlier, be assertive, and to construct substantial defence mechanisms.^108^ Prosser stated that working class families, by contrast, and particularly those receiving state benefits, took a more submissive role, accepted the decisions made by Social Services, and did not seek out a solicitor or a second medical opinion.^109^ Prosser did not explain what indicators he took to represent a ‘middle class’ and a ‘working class’ parent. It seems likely that the parents who Prosser deemed ‘middle class’, who, he stated, were more likely to challenge child protection proceedings, were also more likely to join PAIN. Certainly, Prosser only described one of the families who he visited as ‘working class’.^110^ This chimes with the findings of numerous historians and sociologists that the membership of New Social Movements and non-governmental organizations was usually dominated by the middle classes.^111^

Certainly, the case studies which PAIN offered to media, researchers and policy-makers presented a very specific model of the family. I would contend that PAIN sought to represent the ‘respectable family’. Notions of respectability have long been ill-defined, yet tied to ideas around status and morality.^112^ PAIN families had lost ‘respectability’ because they had been accused of fundamentally transgressing their parental roles, and abusing their children. PAIN sought to reassert the status of these families as ‘respectable’. The case studies offered by PAIN to media and researchers presented families who worked in respected professions, such as law, medicine, and teaching.^113^ The families could afford good solicitors, had never been involved with the police before, and meticulously collected and retained the documents related to their cases.^114^ Prosser described PAIN parents’ homes as ‘well furnished’, ‘comfortable and attractive’, ‘clean and tidy’, and ‘extremely well renovated’, and as located within ‘pleasant’ neighbourhoods or ‘just outside a rural village’. These descriptions held much in common with F.M.L. Thompson’s Victorian ‘respectable working-class homes’.^115^

The presentation of PAIN members as respectable was promoted in the British Broadcasting Corporation (BBC) documentary *Open Space: Innocents at Risk*. Aired on 17 March 1986, *Innocents at Risk* was a half-hour documentary produced by the Community Programming Unit which, between 1972 and 2004, commissioned programmes ‘proposed by people with opinions that are under-represented on television’.^116^ *Innocents at Risk* was made in collaboration between a Director and a Producer from the BBC, Stephanie Cartwright and Gavin Dutton, and PAIN. Television programmes reach an indefinite range of potential recipients, presenting a one-directional form of communication.^117^ The historian Joe Moran has written that the 1970s and 1980s were an ‘age of one-nation television’ as ‘the same programmes were watched and loved by huge and diverse audiences’.^118^ Creating *Innocents at Risk* thus gave PAIN the opportunity to disseminate their key messages to the British public.

The programme featured interviews with the Amphletts and three other families represented by PAIN. The programme opened by portraying a well made up woman wearing a formal dress and standing in a pleasant and well-furnished home. The woman is gazing into a large mirror as she carefully puts on expensive-looking earrings. A man appears, dressed in a smart suit. The man carefully puts on a tie, using the same mirror. The narrator tells us that this is Darren and Helen, a married couple whose baby was taken into care at 6 weeks old under allegations of physical abuse. The parents are preparing to make a court appearance seeking the return of their daughter. The careful positioning of the earrings and the tie provide as a visual signifier of the affluence of the family, and the seriousness and respect with which the family treats a court appearance. The parents’ gestures mirror one another, and they are both reflected in the mirror, accentuating the synchronicity of their lives and their shared determination to seek the return of their daughter. Susan Amphlett told the documentary viewers that, for this type of parent, being placed on the child abuse register put: ‘a wedge through your family life’. PAIN members found the challenge to whether you’re ‘bringing up your family in a responsible way’ ‘beyond words’ and ‘simply not tolerable’.^119^ Through visual message, case study and word, the documentary portrayed a respectable type of parent who would not abuse their children.

Thompson and the sociologist Beverely Skeggs have demonstrated that ideas of respectability have contained clear judgements about gender.^120^ Indeed, Susan Amphlett told the documentary that being involved in child protection investigations made her ‘begin to doubt my capabilities as a mother’.^121^ Helen also emphasized that she felt ‘broken down’ when her child was taken away.^122^ While her daughter was in care Helen felt that their mother–child bond was being eroded, as was physically denoted when Helen could no longer breastfeed her.^123^ Helen was devastated to lose her mothering role, stating that: ‘as far as she was concerned I was just another person, I wasn’t her mother, theoretically the foster mum was her mother, she did everything for her that a mother should do’.^124^ The documentary reveals that Darren and Helen’s child was returned to them by the court. In a subsequent interview Helen expresses joy at having regained not only her child but also her role as a mother. The interviewer asks Helen ‘How are you feeling?’ Helen responds that she is feeling ‘Brilliant … I’m a Mum again’.^125^ Helen’s emotional state is explicitly linked to her ability to perform her role as a mother. Whilst Helen’s testimony to the *Innocents at Risk* documentary is long, detailed, and emotive, her husband Darren is less prominently featured. More broadly, in a report to the Department of Health and Social Security in 1986, PAIN wrote that when placed on the child abuse register, many mothers who were working gave up their jobs ‘for fear that if they carried on working they would be seen as uncaring’.^126^ It is also notable that Susan Amphlett gave up her job as a nurse to organize PAIN, whilst Steven Amphlett did not.

*Innocents at Risk* was viewed by 1.4 million viewers, the largest audience Open Space had had.^127^ To these viewers, PAIN presented a very specific model of the family, which reflected broader social and political anxieties about the family, parenting and particularly motherhood. The construction of PAIN’s respectable families spoke to the concerns of New Right figures and moral crusaders that ‘traditional’ or ‘golden age’ families were being destroyed by rising rates of divorce, illegitimacy and single parenthood, as well as global Fordism, large-scale immigration, and the appearance of ‘problem families’.^128^ PAIN’s focus on the mother as the central figure has a long history, but was also a reflection of psychoanalytic research around ‘attachment theory’ and the importance of maternal love for child development, influential in contemporary parenting manuals and thought, as well as new concerns around the expanding employment of women and emergence of second-wave feminism.^129^ Whilst representing a difficult cause, PAIN sought to benefit from prevalent—if not necessarily well-founded—concerns about the state of the family.

It is unlikely that PAIN’s portrayal of the respectable family represented the experiences of all families who were falsely accused of child abuse. Indeed, other voluntary groups emerged seeking to support and help different ‘types of parents’. The Five Percenters sought to unite, support and consolidate a knowledge-base for parents who were falsely accused of shaking their babies. The groups’ name referenced the belief that one shaken baby case in twenty was misdiagnosed.^130^ Regional groups also, such as those established in Leeds, Cleveland, Orkney and Rochdale, enabled parents from the same locale to meet physically, and to support one another more easily, as well as to exchange specific information about the issues in their region.^131^ The lack of attention paid to fathers facilitated the emergence of fathers rights groups, such as Families Need Fathers (1974), Dads Against Discrimination (2002), Even Toddlers Need Fathers (2003) and Fathers 4 Justice (2002). In 1999, the group Falsely Accused Carers and Teachers emerged seeking to support falsely accused teachers, social workers and physicians.^132^ Parents groups have also emerged in new areas since the late 1990s and early 2000s, reflecting new concerns such as campaigns around vaccinations and autism.^133^

Since the closure of the initial PAIN in 1999, similar groups have not emerged, even though parents continue to be falsely accused of child abuse. It is difficult to understand how to interpret this. Some falsely accused parents are still represented by the new PAIN, although this is a far smaller organization. Why do falsely accused parents no longer unite, mobilize and campaign? With an increasingly tense climate around child protection issues, and attention shifting towards helping survivors, perhaps parents are now less willing to publicly announce that they have been accused of abuse. As I outlined, policy has also shifted towards further including parents in child protection, so perhaps to an extent falsely accused parents feel less need to engage in activism, and are looking for solutions within ‘the system’. It is possible that falsely accused parents are still united, but online within chat rooms, and via email, with new and private technologies which are not visible to the historian. On the other hand, perhaps the barriers to activism appear higher to falsely accused parents, amidst the professionalization and competitiveness of the voluntary sector.

The landscape of activism has thus changed in response to shifting social and moral concerns, as well as to reflect the existing foci of the voluntary sector and policy, and the shape of PAIN’s campaigning speaks to anxieties around the family, parent and the child in 1980s and 1990s Britain. PAIN was a product not only of the experiences of the Amphletts, but also of a historical period imbued with anxieties around family life. Like any voluntary group, PAIN could not represent everyone, despite relying on experience to work effectively with parents and policy-makers. Nonetheless, the group did work with a substantial number of parents, who understood and lived their experiences through the mediation and publications of this group.

## Conclusion

The history of late-twentieth-century Britain remains incomplete without attention to the small voluntary groups who acted as ‘buffers’ between new identity-constituencies and traditional sources of ‘expertise’ such as physicians, social workers, solicitors, and policy-makers. These groups have emerged and multiplied in the field of child protection since the 1970s. Alongside PAIN, groups have also emerged to support, represent and empower adults who were abused as children, including national groups such as the National Association for People Abused in Childhood, One in Four UK and Phoenix Survivors, and also regional groups including Survivors Swindon, Survivors Helping Each Other, Nottinghamshire, and Norfolk’s Surviving Together. Buffer groups emerge because child protection and child abuse are serious, important and emotive issues which straddle institutions related to health, crime and welfare. Many other issues—such as drug use, sexuality, juvenile crime and homelessness—also straddle such areas, however, and it is likely that small groups have emerged to act as buffers in these areas too.

These groups are not without precedent—since the sixteenth-century fraternities and religious guilds have provided space for mutual aid and self-help, traditions continued into the nineteenth century by Friendly Societies and the Co-operative Movement, and in the twentieth century by trade unionism.^134^ Whilst PAIN had historical precedents, much of its activism was characteristic of the post-war period. Parents were able to communicate with one another utilizing developing forms of communication technology, particularly the telephone line, also adopted in the post-war period by the Samaritans (since 1953), Britain’s Gay Switchboard (founded 1974), and ChildLine (1986).^135^ Parents shared their emotions with this broader collective—telling deeply personal stories within the organization’s newsletters, and in newspaper interviews and through a television documentary, which supports the assertions of Deborah Cohen and Adrian Bingham that the post-war period saw a broader ‘confessional culture’, whereby adults increasingly described and revealed experiences which would have previously been kept secret. Cohen and Bingham point towards agony aunt columns, newspaper reporting, and the boom in counselling, psychiatry and psychoanalysis, but voluntary groupings were also formed around new issues of identity.^136^ Indeed, ‘identity politics’ developed and extended in the 1960s, 1970s and 1980s, amidst a shift from material to life and identity concerns. New social issues—such as child abuse—brought new identities, such as the falsely accused parent.

In the post-war period, the state has expanded and extended to new forms of intervention in education, welfare and health. The campaigning of PAIN points towards the broader and concurrent idea that representatives of the public should be able to contribute to policy-construction, seen in literatures around how patients and ‘service users’ were reconfigured from ‘passive’ to ‘active’ recipients of welfare in the 1980s, 1990s and 2000s.^137^ This trend has continued in child protection, in rhetoric if not in reality, as may be seen by Home Secretary Theresa May’s determination to gain the ‘confidence of survivors’ who must have a ‘strong voice’ and be ‘at the heart’ of the Independent Inquiry into Child Abuse, first established in July 2014 in order to ‘consider whether public bodies—and other, non-state, institutions—have taken seriously their duty of care to protect children from sexual abuse’.^138^

PAIN was able to work with parents because the Amphletts had personal experience of being falsely accused, and also gained professional experience in working on child protection cases. PAIN was able to work with policy-makers because the group collated and brought personal experiences to bear on political issues, through the traditional routes of letter-writing and contributing to inquiries, and also through the media. PAIN thus combined the passion and identity-claims of New Social Movements and service-user organizations, and the technocratic expertise of non-governmental organizations. The existence and relative successes of PAIN suggest that experiential and professional knowledge were entwined in the politics of the late twentieth century. PAIN was a very small group, but nonetheless acted as a buffer within the intermediary terrain between public and expert, negotiating and shaping ideas of parenting, family, childhood, respectability and the state.

## Footnotes

^1^ Bodleian Library (Hereafter Bod), Oxford, M89.B00525, Susan Amphlett, ‘Susan Amphlett's statement to the Cleveland Inquiry', 14 December 1987, 1.

^2^ Modern Records Centre (Hereafter MRC), Coventry, MSS.378/BASW/7/28, Susan and Steve Amphlett, ‘PAIN—Parents Against Injustice Publicity Material', Untitled and Undated Statement, 2.

^3^ MRC, Amphlett and Amphlett, ‘Publicity Material', 2; Bod, Amphlett, ‘Statement to the Cleveland Inquiry', 1.

^4^ David Brindle, ‘Painful Departure', *Guardian*, 14 April 1999, 6; Victor Smart, ‘Parents' Group Gets Government Grant', *Guardian*, 5 July 1987, 4; James Erlichman, ‘Ordeal of the Innocent: of Course the Young Must be Protected, but …', *Guardian*, 10 June 1997; Parents Against Injustice, ‘About Us' <http://parentsagainstinjustice.org.uk/about.html> accessed 20 May 2015.

^5^ Erlichman, ‘Ordeal of the Innocent', 17; Will Wyatt, ‘Kennedy, Sir Ludovic Henry Coverley (1919-2009)', *Oxford Dictionary of National Biography* (January 2013) <http://www.oxforddnb.com/templates/article.jsp?articleid=101900&back> accessed 25 June 2014.

^6^ British Library (hereafter BL), ZK.9.b.1682, Parents Against Injustice Newsletters, Newsletter 1: January 1987, ‘General Account', 8; BL, ZK.9.b.1682, Parents Against Injustice Newsletters, Newsletter 2: July 1987, ‘General Account', 22-3; BL, ZK.9.b.1682, Parents Against Injustice Newsletters, Newsletter 6: August 1989, ‘Income and Expenditure Account', Insert to booklet.

^7^ BL, Parents Against Injustice Newsletters, Newsletter 2: July 1987, ‘….And Now, for My Next Trick', Noelene, 21.

^8^ Brindle, ‘Painful Departure', 6.

^9^ Richard Ingrams, ‘Catholics-R-Us; Plus Some Rather Peculiar Conduct on the Buses', *The Observer*, 7 July 1996, 62.

^10^ Michael Simmons, ‘Matters Arising', *Guardian*, 24 July 1996, 86.

^11^ Brindle, ‘Painful Departure', 6.

^12^ House of Commons Education Select Committee, *Children first: the child protection system in England*, Written evidence submitted by Parents Against Injustice (PAIN), November 2011, <http://www.publications.parliament.uk/pa/cm201213/cmselect/cmeduc/137/137vw24.htm> accessed 10 August 2014; House of Commons Children, Schools and Families Select Committee, *Looked-after Children*, Third Report of Session 2008-9, Volume II, Oral and Written Evidence (London, 2009), 210.

^13^ Parents Against Injustice <http://www.parentsagainstinjustice.org.uk> accessed 10 August 2014; Parents Against Injustice <http://parentsagainstinjustice.ning.com/> accessed 10 August 2014.

^14^ Peter Davenport, ‘Parents Fight “Abuse” Cases', *The Times*, 11 April 1988, 3; James J Clyde, *The Report of the Inquiry into the Removal of Children from Orkney in February 1991* (London, 1992), 126; Tom Sharratt, ‘Top Judge to Preside in Child Abuse Case', *Guardian*, 20 September 1990, 3.

^15^ Gerard Seenan, ‘Parents Fight “Shaken Baby” Stigma', *Guardian*, 18 August 1998, 8.

^16^ Phillip Jenkins, *Intimate Enemies: Moral Panics in Contemporary Great Britain* (New York, 1992), 135, 137.

^17^ Jenkins, *Intimate Enemies*, 140–1.

^18^ TNA, BN 68/15, Inquiry into Child Abuse in Cleveland 1987: Report and Papers, Evidence and Papers Submitted to the Inquiry, Parents and Cleveland Parents' Support Group: Statements from Reverend Michael Wright (Group Co-ordinator), 13 August 1987, 1, 4.

^19^ Nigel Parton, *The Politics of Child Protection: Contemporary Developments and Future Directions* (Basingstoke, 2014) 21.

^20^ Louise Jackson, *Child Sexual Abuse in Victorian England* (London, 2000) 3–4; Linda Gordon, *Heroes of their own Lives: The Politics and History of Family Violence* (London, 1989), 32–3; George Behlmer, *Child Abuse and Moral Reform in England* (Stanford, 1982), 16.

^21^ Ian Hacking, *Social Construction of What?* (Cambridge, MA, 1999), 125–6.

^22^ Angela Neustatter, ‘For the Sake of the Innocents: The Balance Between the Rights of Wronged Children, and those of Wrongly Accused Parents, is Never Easy to Achieve. When the Professionals Get it Wrong, the Whole Family May Suffer', *Guardian*, 5 July 1988, 16; Erlichman, ‘Ordeal of the Innocent', 17.

^23^ Jenkins, *Intimate Enemies*, 143–4; Parton, *The Politics of Child Protection*, 24.

^24^ Deborah Cohen, *Family Secrets: Living with Shame from the Victorians to the Present Day* (London, 2013).

^25^ Frank Prochaska, *Christianity and Social Service in Modern Britain: The Disinherited Spirit* (Oxford, 2006), 97.

^26^ Matthew Hilton, James McKay, Nicholas Crowson, and Jean-Francois Mouhot, ‘The Big Society: Civic Participation and the State in Modern Britain', *History **and **Policy*, 8 June 2010 <http://www.historyandpolicy.org/policy-papers/papers/the-big-society-civic-participation-and-the-state-in-modern-britain>.

^27^ Alex Mold, ‘“The Welfare Branch of the Alternative Society?” The Work of Drug Voluntary Organization Release, 1967-1978’, *Twentieth Century British History*, 17 (2006), 52.

^28^ Mold, The Welfare Branch of the Alternative Society? 72.

^29^ Virginia Berridge and Alex Mold, *Voluntary Action and Illegal Drugs. Health and Society in Britain Since the 1960s *(Basingstoke, 2010), 147.

^30^ MRC, Amphlett and Amphlett, ‘Untitled and Undated Statement', 1.

^31^ MRC, Amphlett and Amphlett, ‘Publicity Material', 1.

^32^ MRC, 1.

^33^ MRC, 1.

^34^ MRC, 2; Susan Amphlett, ‘Child Testimonies and Innocent Parents', *Independent*, 27 October 1987, 19.

^35^ BOD ‘Susan Amphlett's Statement to the Cleveland Inquiry', 1.

^36^ BL, Parents Against Injustice Newsletters, Newsletter 3: August 1988, ‘Have You Ever Wondered….?', 19.

^37^ BL, 19.

^38^ BL, 19.

^39^ BL, Parents Against Injustice Newsletters, Newsletter 3: August 1988, Noelene, ‘…And Now, for My Next Trick', 21.

^40^ Erlichman, ‘Ordeal of the Innocent', 17.

^41^ Smart, ‘Parents' Group Gets Government Grant', 4; Erlichman, ‘Ordeal of the Innocent', 17.

^42^ Sue Amphlett, ‘The Experience of a Watchdog Group', in Geoffrey Hunt, ed., *Whistleblowing in the Social Services: Public Accountability and Professional Practice* (London, 1998), 66.

^43^ BL, Parents Against Injustice Newsletters, Newsletter 2: July 1987, ‘Dear Members' Susan Amphlett, 1–2.

^44^ Grace McLean, ‘Doctor Who Put “Abuse” Down to Brittle Bones May be Banned', *Daily Mail*, 15 November 2003, 17.

^45^ BL, Parents Against Injustice Newsletters, Newsletter 2: July 1987, ‘Dear Members' Susan Amphlett, 1–2.

^46^ BL, Parents Against Injustice Newsletters, Newsletter 3: August 1988, 2; BL, Parents Against Injustice Newsletters, Newsletter 6: August 1989, 1–6.

^47^ BL, Parents Against Injustice Newsletters, Newsletter 1: January 1987, ‘Training Programmes', 8.

^48^ BL, 1–6.

^49^ BL, 1–6.

^50^ BL, Parents Against Injustice Newsletters, Newsletter 6: August 1989, ‘Regional Administrators Reports', 5.

^51^ BL, Parents Against Injustice Newsletters, Newsletter 6: August 1989, ‘South and South West', 2; BL, Parents Against Injustice Newsletters, Newsletter 6: August 1989, ‘Midland Region', 6.

^52^ Laura Topham, ‘Inspirational Women of the Year: Alison's Son was Snatched Away After She was Wrongly Branded a Child Abuser … Now She Fights for Justice for Other Mothers', *Daily Mail*, 16 April 2009 <http://www.dailymail.co.uk/femail/article-1170370/Inspirational-women-Alison-wrongly-branded-child-abuser-fights-justice-others.html> accessed 20 May 2015; Babita Wakelin, ‘We Were Victims of Child Snatchers', *Leicester Mercury*, 1 June 2005, 10.

^53^ BL, Parents Against Injustice Newsletters, Newsletter 6: August 1989, ‘Midland Region', 6.

^54^ BL, Parents Against Injustice Newsletters, Newsletter 1: January 1987, ‘Training Programmes', 8.

^55^ BL, Parents Against Injustice Newsletters, Newsletter 6: August 1989, ‘Regional Administrators Reports', 4–5.

^56^ BL, Parents Against Injustice Newsletters, Newsletter 3: August 1988, 2; BL, Parents Against Injustice Newsletters, Newsletter 6: August 1989, ‘Regional Administrators', 1.

^57^ BL, Parents Against Injustice Newsletters, Newsletter 6: August 1989, ‘Regional Administrators Reports', 5; BL, Parents Against Injustice Newsletters, Newsletter 6: August 1989, ‘Midland Region', 6.

^58^ BL, Parents Against Injustice Newsletters, Newsletter 6: August 1989, ‘Regional Administrators Reports', 1–2.

^59^ Jane Hoyal et al, ‘Funds Crisis Ends “Unique” Charity', *The Times*, 25 November 1999, 29.

^60^ BL, Parents Against Injustice Newsletters, Newsletter 3: August 1988, 1; BL, Parents Against Injustice Newsletters, Newsletter 6: August 1989, ‘A Trustee's Comment', 6–7.

^61^ BL, Parents Against Injustice Newsletters, Newsletter 1: January 1987, ‘General Account', 8.

^62^ *Parliamentary Debates* (Commons), 155, 30 June 1989, 575; *Parliamentary Debates* (Commons), 171, 24 April 1990, 163; *Parliamentary Debates* (Commons), 191, 21 May 1991, 438; *Parliamentary Debates* (Commons), 227, 21 June 1993, 38.

^63^ BL, Parents Against Injustice Newsletters, Newsletter 1: January 1987, ‘Finances', 9; BL, Parents Against Injustice Newsletters, Newsletter 3: August 1988, ‘Enrolment Form', 9.

^64^ BL, Parents Against Injustice Newsletters, Newsletter 2: July 1987, ‘Equipment', 2; BL, Parents Against Injustice Newsletters, Newsletter 2: July 1987, ‘Dear Members' Susan Amphlett, 1–2.

^65^ BL, Parents Against Injustice Newsletters, Newsletter 1: January 1987, ‘Why was PAIN Formed?', 1.

^66^ BL, Parents Against Injustice Newsletters, Newsletter 1: January 1987, Nigel Parton, ‘Foreword', 3.

^67^ Anthea Gerrie, ‘Victims of the Abuse “Experts”', *Daily Mail*, 24 June 1987, 12; Anthea Gerrie, ‘How Can They Call This “Care”?', *Daily Mail*, 4 July 1987, 6.

^68^ Jon Prosser, *Child Abuse Investigations: The Families' Perspective: A Case Study of Thirty Families Who Claim to Have Been Falsely Accused *(Essex, 1992), 1; Sharratt, ‘Top Judge to Preside in Child Abuse Case', 3; Children, Schools and Families Committee, *Looked-after Children*, 210.

^69^ Gerrie, ‘Victims of the Abuse “Experts”', 12.

^70^ MRC, Parents Against Injustice Publicity Material, Untitled Letter by Susan and Steve Amphlett, Undated; Jane Hodgkin, ‘Parents Against Injustice', *New Society*, 16 May 1986, 5–6; Howard Sharron, ‘Parent Abuse', *New Society*, 13 March 1987, 22; BOD ‘Susan Amphlett's Statement to the Cleveland Inquiry', 26.

^71^ Michel Foucault, *Discipline and Punish: the Birth of the Prison* (2nd edn, New York, 1995), 211–16.

^72^ Michael Simmons, ‘Matters Arising', *Guardian*, 24 July 1996, 86; Sharron, ‘Parent Abuse’, 22.

^73^ MRC, Parents Against Injustice Publicity Material, Untitled Pamphlet, Undated; British Film Institute (hereafter BFI), LCPW008X, *Open Space: Innocents at Risk*, Dir. Stephanie Cartwright, 17 March 1986; Gerrie, ‘How Can They Call This “Care”?', 6; Nicola Tyrer and Chris Tighe, ‘The Cleveland Dilemma', *Irish Independent*, 26 June 1987, 8; BL, Parents Against Injustice, *A response to Child Abuse**—**Working Together*, 35; BL, Parents Against Injustice Newsletters, Newsletter 1: January 1989, ‘Dear Members', Susan Amphlett, 1–2.

^74^ MRC, Amphlett and Amphlett, Untitled Pamphlet; BFI, *Open Space*; Gerrie, ‘How Can They Call This “Care”?', 6; MRC, Amphlett and Amphlett, ‘Untitled Letter'. At a similar time, the name of Ratepayers Against the Greenham Encampments (RAGE) sought to encapsulate the group's ‘combative intent' (Christopher Moores, ‘Opposition to the Greenham Women's Peace Camps in 1980s Britain: RAGE Against the “Obscene”', *History Workshop Journal* (2014), 1.)

^75^ Gerrie, ‘How Can They Call This “Care”?', 6.

^76^ BL, Parents Against Injustice Newsletters, Newsletter 1: January 1987, ‘Case Histories', 6.

^77^ BL, Parents Against Injustice Newsletters, Newsletter 1: January 1987, ‘Case Histories', 6–7.

^78^ Erlichman, ‘Ordeal of the Innocent', 17.

^79^ BOD, Amphlett, ‘Statement to the Cleveland Inquiry', 3, 14–17.

^80^ BOD, 1.

^81^ BOD, 1.

^82^ BL, Parents Against Injustice Newsletters, Newsletter 1: January 1987, ‘How PAIN has Grown', 5–6.

^83^ BL, Parents Against Injustice Newsletters, Newsletter 3: August 1988, ‘Statements from the New Trustees', 3.

^84^ BL, Parents Against Injustice Newsletters, Newsletter 2: July 1987, ‘Editor's Note', 19.

^85^ BL, Parents Against Injustice Newsletters, Newsletter 1: January 1987, ‘How PAIN has Grown', 5–6.

^86^ BL, YC.1989.b.433, ‘Innocents at Risk', Susan and Steve Amphlett, 1986, 18.

^87^ Ingrams, ‘Catholics-R-Us', 62.

^88^ BOD, 26.

^89^ ‘Smoke without fire', *Guardian*, 12 January 2000 <http://www.theguardian.com/lifeandstyle/2000/jan/12/familyandrelationships.features10> accessed 17 February 2015.

^90^ Ingrams, ‘Catholics-R-Us', 62; Simmons, ‘Matters Arising', 86.

^91^ Gerrie, ‘How Can They Call This “Care”?', 6.

^92^ Prosser, *Child **Abuse **Investigations*, 8; Peter Dale, ‘Like a Fish in A Bowl: Parents' Perceptions of Child Protection Services', *Child Abuse Review*, 13 (2004), 138.

^93^ HM Government, *The Children Act 1989: Introduction*, Cm 2144, (London, 1989), 3.

^94^ HM Government, *The Children Act 1989*, Section 45: Emergency protection orders and other supplemental provisions.

^95^ HM Government, *The Children Act 1989*, Section 45 and Section 26: Review of cases and enquiries into representations.

^96^ HM Government, *The Children Act 1989*, Section 45 and 26.

^97^ Brindle, ‘Painful Departure', 6; Children, Schools and Families Committee, *Looked-after Children*, 210.

^98^ Children, Schools and Families Committee, *Looked-after Children*, 210.

^99^ Education Committee, *Children **First*, Fourth Report of the Session 2012-2013, Volume III: Additional written evidence, 30 October 2012, 74–5.

^100^ Conservative Party, *1997 Conservative Party General Election Manifesto: You can only be sure with the Conservatives* <http://www.conservativemanifesto.com/1997/1997-conservative-manifesto.shtml> accessed 20 May 2015.

^101^ Kate Wilson and Adrian James, *The Child Protection Handbook: The Practitioner's Guide to Safeguarding Children* (London, 1995), 14.

^102^ Brindle, ‘Painful Departure', 6.

^103^ Brindle, ‘Painful Departure', p. 6; ‘Smoke without Fire', *Guardian*, 12 January 2000 <http://www.theguardian.com/lifeandstyle/2000/jan/12/familyandrelationships.features10> accessed 17 February 2015.

^104^ Ann Macaskill and Peter Ashworth, ‘Parental Participation in Child Protection Case Conferences: The Social Worker's View', *British Journal of Social Work*, 25 (1995) 582; June Thoburn and David Shemmings, *Parental Participation in Child Protection Conferences: Report of a Pilot Study in Hackney Social Services Department* (Norwich, 1990).

^105^ Ray Jones, ‘Children's Acts 1948-2008: the Drivers for Legislative Change in England over 60 Years', *Journal of Children's Services*, 4 (2009), 39–52.

^106^ Rosie Waterhouse, ‘Innocent Suffer during Inquiries into Child Abuse', *Independent*, 1 December 1992, 8; Peter Smallridge, ‘Distorted Picture of Child-Abuse Inquiries', *Independent*, 7 December 1992, 16; Sue Amphlett, ‘Protecting Children from Abuse', *Independent*, 9 December 1992, 24; Marion Bennathan and Robert Laslett, ‘Victimised by False Allegations of Abuse', *Independent*, 12 December 1992, 17.

^107^ BL, Parents Against Injustice Newsletters, Newsletter 3: August 1988, ‘Statements from the New Trustees', 3; BL, Parents Against Injustice Newsletters, Newsletter 6: August 1989, ‘A Trustee's Comment', 6–7.

^108^ Prosser, 15–16.

^109^ Prosser, 15–16.

^110^ Prosser, 56.

^111^ See essays by Caitriona Beaumont, Tanya Evans, Lawrence Black, and Christopher Rootes in Nick Crowson, Matthew Hilton and James McKay, eds, *NGOs in Contemporary Britain: Non-State Actors in Society and Politics since 1945 *(Basingstoke, 2009); Frank Parkin, *Middle Class Radicalism: The Social Bases of the British Campaign for Nuclear Disarmament* (Manchester, 1968).

^112^ F.M.L. Thompson, *Rise of the Respectable Society: A Social History of Victorian Britain, 1830-1900* (Cambridge, MA, 1990), 181; Beverley Skeggs, *Formations of Class & Gender: Becoming Respectable* (London, 2001), 1–2.

^113^ Gerrie, ‘Victims of the Abuse “Experts”', 12.

^114^ Gerrie, ‘Victims of the Abuse “Experts”', 12; Prosser, *Child **Abuse **Investigations*, 64; BFI, *Open Space*.

^115^ Prosser, *Child **Abuse **I**nvestigations*, 25, 28, 47, 64.

^116^ Richard Kilborn and John Izod, *An Introduction to Television Documentary: Confronting Reality* (Manchester, 1997), 82–3.

^117^ John Thompson, ‘The New Visibility', *Theory, Culture and Society*, 22 (2005), 33.

^118^ Joe Moran, *Armchair Nation: An Intimate History of Britain in Front of the TV* (London, 2013), 2.

^119^ BFI, *Open Space*.

^120^ Skeggs, *Formations of Class & Gender*, 1–2.

^121^ BFI, *Open Space*.

^122^ BFI, *Open Space*.

^123^ BFI, *Open Space*.

^124^ BFI, *Open Space*.

^125^ BFI, *Open Space*.

^126^ BL, Parents Against Injustice, *A Response to Child Abuse**—**Working Together*, 36.

^127^ British Broadcasting Corporation Written Archives, BARB Viewing Figures 17 March 1986, 1; BL, Parents Against Injustice Newsletters, Newsletter 1: January 1987, ‘How PAIN has Grown', 5.

^128^ Andrew Gamble, *The Free Economy and the Strong State: the Politics of Thatcherism* (Basingstoke, 1994); Pat Thane, ‘Family Life and “Normality” in Postwar British Culture’, in Richard Bessel and Dirk Schumann, eds, *Life After Death. Approaches to a Cultural and Social History of Europe during the 1940s and 1950s *(Cambridge, 2003), 193–210; John Welshman, ‘“Troubled Families”: the Lessons of History, 1880-2012', *History **an**d Policy* (1 October 2012) <http://www.historyandpolicy.org/policy-papers/papers/troubled-families-the-lessons-of-history-1880-2012> accessed 20 July 2014.

^129^ Rima Apple, ‘Constructing Mothers: Scientific Motherhood in the Nineteenth and Twentieth Centuries', *Social History of Medicine*, 8 (1995), 161–78; John Bowlby, ‘The Nature of the Child's Tie to his Mother', *International Journal of Psychoanalysis*, 39 (1958), 350–73; John Bowlby, *Maternal Care and Mental Health* (Geneva, 1951); Michal Shapira, *The War Inside: Psychoanalysis, Total War, and the Making of the Democractic Self in Postwar Britain* (Cambridge, 2013), 16–17, 198–238.

^130^ Seenan, ‘Parents Fight “Shaken Baby” Stigma', 8; Deborah Orr, ‘Who Would Harm our Baby?', *Guardian*, 11 May 2013, 54.

^131^ Davenport, ‘Parents Fight “Abuse” Cases', 3; James Clyde, *The Report of the Inquiry into the Removal of Children from Orkney in February 1991* (London, 1992), 126; Sharratt, ‘Top Judge to Preside in Child Abuse Case', 3.

^132^ Falsely Accused Carers and Teachers, ‘Our Origins' <http://www.factuk.org/about-us/our-origins/> accessed 30 May 2015.

^133^ Falsely Accused Carers and Teachers, ‘Our Mission' <http://www.arnica.org.uk/our-mission> accessed 30 May 2015.

^134^ Mold and Berridge, *Voluntary Action and Illegal Drugs, *7, 136; Justin Davis Smith, ‘The Voluntary Tradition: Philanthropy and Self-Help in Britain 1500-1945', in Justin Davis Smith, Colin Rochester and Rodney Hedley, eds, *An Introduction to the Voluntary Sector* (London, 1995), 10–11, 27–30.

^135^ Hereward Harrison, ‘Childline—the First Twelve Years', *British Medical Journal*, 82 (2000), 283; ‘Switchboards, Gay', George E. Haggerty, ed., *Gay Histories and Cultures *(Abingdon, 2012), 856.

^136^ Cohen, *Family Secrets*, 75–76, 196; Adrian Bingham, *Family Newspapers? Sex, Private Life and the British Popular Press 1918-1978* (Oxford, 2009).

^137^ Berridge and Mold, *Voluntary Action and Illegal Drugs*, 147.

^138^ Theresa May, ‘Written Statement to Parliament: Child Sexual Abuse (Woolf Inquiry)', 21 October 2014 <https://www.gov.uk/government/speeches/child-sexual-abuse-woolf-inquiry> accessed 20 May 2015.

